# Patterns of sedentary behavior among older women with urinary incontinence and urinary symptoms: a scoping review

**DOI:** 10.1186/s12889-024-18703-7

**Published:** 2024-04-30

**Authors:** Wilson Kin Chung Leung, Jasmine Cheung, Vivian Chin Ching Wong, Kelly Ka Lee Tse, Ruby Wing Yin Lee, Simon Ching Lam, Lorna Kwai Ping Suen

**Affiliations:** 1 School of Nursing, Tung Wah College, 16/F, Ma Kam Chan Memorial Building, 31 Wylie Road, Kowloon, Hong Kong SAR, China; 2 School of Arts and Humanities, Tung Wah College, 17/F, Cheung Kung Hai Memorial Building, 90A Shantung Street, Kowloon, Hong Kong SAR, China

**Keywords:** Urinary incontinence, Overactive bladder, Lower urinary tract symptoms, Sedentariness, Sedentary lifestyle, Aged, Elderly, Older female

## Abstract

**Background:**

Independent of physical activity, sedentary behavior has emerged as a significant risk factor for health. Particularly, older adults spent as high as 13 h daily on sedentary activities, which account for 98% of their awake times. Although there is growing evidence revealing the potential association between sedentary behavior and urinary incontinence (UI) across populations of different ages, the relationship between sedentary behavior and urinary symptoms in older women, who are twice as likely to have UI than older men, has not been reviewed. This scoping review aimed to synthesize available evidence of the relationship between sedentary behavior and urinary symptoms in noninstitutionalized older women.

**Methods:**

Six electronic databases (PubMed, Web of Science, SPORTDiscus, Ovid Nursing Database, EMBASE, and MEDLINE) were searched from their inception to April 2023. Observational and experimental studies that measured sedentary behavior using objective and/or self-reported methods in older women aged 60 + years having any type of UI, with English full texts available, were included. Relevant data, including sedentary patterns (types, definitions, measurements, and daily patterns) and UI types were tabulated. A narrative synthesis of the findings was also conducted.

**Results:**

A total of seven studies (*n* = 1,822) were included for review and reporting. Objective measurement showed that older women with UI were engaged in > 8 h sedentary activities daily (493.3–509.4 min/day), which accounted for 73% of their awake times. The duration of self-reported sedentary behavior was lower than the time measured objectively, and the average weekday sitting time was 300–380 min/day. With or without adjustment for confounding factors (e.g., age and number of vaginal deliveries), the daily proportion of sedentary time and average duration of sedentary bouts were positively associated with the prevalence of urgency UI. Notably, sedentary patients with UI were more likely to have lower urinary tract symptoms, including bothersome incontinence, to use incontinence products, and to have nocturia episodes, than their age-matched counterparts who were less sedentary.

**Conclusion:**

Our findings suggest a potential relationship between sedentary behavior and UI in older women, but the causality of the relationship remains unclear. To further inform the clinical role of sedentary behavior in the context of UI, a greater number of rigorous studies with a prospective study design is urgently needed.

**Supplementary Information:**

The online version contains supplementary material available at 10.1186/s12889-024-18703-7.

## Background

Urinary incontinence (UI), also known as involuntary urination, represents uncontrolled leakage of urine [1]. UI has six types, namely, stress, urgency, mixed, overflow, functional, and reflex, and stress UI (a leakage of urine during movement or physical activity that causes an increment in abdominal pressure, such as coughing, laughing, sneezing, exercising, or heavy lifting), urgency UI (caused by a sudden and intense urge to urinate), and a mixture of both are the most common types. This condition occurs in both sexes but affects twice as many women as men [2–6]. Similar to accidental falls, UI is regarded as one of the most prevalent geriatric syndromes, particularly in institutionalized older people [7].

In women, an increased risk of UI is associated with advanced age, pregnancy, and parity status [3]. Approximately 40% of women aged ≥ 70 years are affected [8], and pregnancy and childbirth are associated with 30%–40% increase in the occurrence of UI [3]. Other significant risk factors that may predispose women to UI include excess body weight, which increases abdominal pressure, estrogen deficiency, which occurs during menopause and weakens the urethra, pelvic floor muscles, and abdominal muscles, as well as activities performing domestic tasks that were usually omitted from questionnaires [3, 9, 10]. Despite that UI is a common problem for women, it is usually under-reported by incontinent women because of embarrassment or misconception that UI is a normal part of ageing [11]. Therefore, only 15%–25% of women who were incontinent voluntarily sought professional help for UI, and thousands of them had not been diagnosed or appropriately treated. As they might avoid social situations or travel due to “embarrassing” condition, their psychosocial health is eventually negatively influenced (lower self-esteem, depressed mood, and increased stress) [11, 12].

Currently, behavioral interventions (e.g., exercise, diet, or a combination of both) have been proposed as promising strategies for relieving the burden of overall UI across various at-risk female populations with clinical conditions, such as excess weight (obesity) [13, 14] and pregnancy [15, 16]. A 5%–10% weight loss induced by a behavioral program involving diet and exercise led to a significant decrease in UI episodes by 30% in overweight women [14]. During pregnancy and postpartum period, women should be encouraged to engage in an active lifestyle involving aerobic and muscle-strengthening activities to prevent the occurrence of UI [15, 16]. Notably, no associations between regular exercise prescribed at an appropriate dosage and unfavorable neonatal outcomes, such as low birth weight and preterm delivery, has been found [16]. Owing to “embarrassing” urine leakage, avoidance of social situations is usually perceived as a barrier that impedes their ability to be physically active [11]. Although habitual physical activity is effective in preventing and managing UI as a result of ageing [17], how a sedentary lifestyle that has a clinically meaningful adverse impact on health [18] affects the risk and severity of UI in older women remains largely unknown. Given that physiological and cognitive response patterns vary between a sedentary and active lifestyle [19], the exact nature of sedentary behavior and its association with UI should be explored.

Sedentary behavior, a movement behavior construct distinct from physical activity, is defined as “any waking behavior characterized by an energy expenditure ≤ 1.5 metabolic equivalent, while in a sitting, reclining or lying posture” [20]. Screen time (TV viewing) and sitting time are often the two major parameters used to indicate the time spent sedentary in older adults [19, 21]. Highly active people can be highly sedentary [22]. For instance, an active individual might participate in ≥ 150 min weekly of moderate-intensity physical activity (e.g., brisk walking or jogging) but spend 16 waking hours a day sitting across different contexts (leisure, transport, and occupation). Independent of physical activity levels, sedentary time is positively associated with disability in daily activities in older adults, and risk increases by 46% for each hour spent sedentary [23]. Commonly, there are two main categories of methods used for measuring sedentary behavior, including objective (e.g., ActivPAL, ActiGraph, or naturalistic observation) and subjective (e.g., self-reported questionnaires) measurements. The subjective measurement of sedentary behavior usually has questionable criterion validity, especially in older adults, because of social desirability bias and cognitive-reliant nature [24–26]. By contrast, objectively measured data may not reflect the context where sedentary time is accumulated (e.g., watching TV, computer use, reading, and socializing) [18, 21, 27].

Findings on objectively measured sedentary behavior showed that independent-living seniors spent approximately 9 h/day sedentary, which equates to 65%–80% of their waking time [28]. In older adults residing in care facilities, daily sedentary time can be as high as 98% of their waking hours (i.e., ~ 13 h a day) because of functional impairments and lack of motivation to join activities [21]. Increased sedentary time, especially > 8 h/day of sitting, has a dose–response relationship with all-cause mortality; the risk of all-cause mortality increased by 31% and 47% for physically-inactive older people who spent 8–11 or > 11 h daily on sitting, respectively [18]. Conversely, restricted sedentary behavior or interrupted sedentary bouts are protective against all-cause mortality and metabolic syndrome (especially central obesity). However, insights gained from theses systematic reviews are limited because no emphasis has been placed on deleterious health effects attributed to sedentary behavior stratified by gender; men and women are inherently different with respect to biological structure and function [29], physical activity determinants [30], and health effects derived from movement or nonmovement behaviors [29, 31].

Evidence of the association between sedentary behavior and UI across populations of different ages has grown [4, 25, 26, 32]. Thus, we conducted a scoping review about this growing research area to investigate potential associations between sedentary behavior and UI in older women.

## Methods

The study protocol was constructed in line with the Arksey and O’Malley framework [33]. The findings were reported in accordance with the Preferred Reporting Items for Systematic Reviews and Meta-Analyses (PRISMA) Extension for Scoping Reviews [34].

### Eligibility criteria

The inclusion and exclusion criteria were set with reference to previously described criteria [21]. Observational (e.g., cross-sectional, case–control, and cohort) and experimental studies were included. For experimental studies, only baseline data for either total participants or participants in the experimental arm were included. The inclusion criteria were as follows: (1) text availability: full text; (2) language: English; (3) article type: peer-reviewed original research; (4) participants: older women aged 60 + years with UI; and (5) study objective: the included studies did not necessarily consider sedentary behavior as the primary outcome, but sedentary behavior (e.g., daily sedentary time, frequency, and duration of sedentary bouts) can be reported as part of the results (i.e., as a covariate). Studies involving older men and women were also considered, but only data on women were included. Given that the risk of having UI is 2.6-fold that during menopause because of decline in estrogen production (i.e., hypoestrogenism) [35], studies involving postmenopausal women who were below 60 years were also included. Studies with results not stratified by age (i.e., not allowed to identify women aged 60 years and older) or only involved male participants, and studies other than original research, including reviews, meta-analyses, study protocols, perspective papers, editorials, letters to the editor, published errata, and commentaries were excluded. Conference abstracts that were usually not peer-reviewed or studies in purely qualitative design that were not in line with our research objectives on identifying sedentary patterns (e.g., daily time, bout durations, and frequency) were also excluded.

### Information sources

Six electronic databases, namely PubMed, Web of Science, SPORTDiscus, Ovid Nursing Database, EMBASE, and MEDLINE, were searched from their inception to April 2023. A manual search of the bibliographies for articles extracted for full-text assessment and existing review articles was carried out to identify potentially eligible studies not captured in the electronic database search.

### Search strategy

The text word terms used in the electronic database search (Title/Abstract/Subject/Keywords) were as follows: (“urinary incontinence” OR “bladder incontinence” OR enuresis OR bed?wetting) and (sedentar* OR “TV viewing” OR “TV watching” OR television OR smartphone OR sitting OR reclining OR lying). To maintain breadth of coverage as our previously described [36], the database search was piloted twice in order to generate a broad range of text word terms used in the search. Also, no restrictions were placed on the target participants in the search, and hence studies that were conducted in older women were manually identified. This highly sensitive search strategy ensured the inclusion of potentially eligible studies to avoid missing of potentially relevant studies [37]. An additional file shows the search queries for the six electronic databases with limiters (see Additional file 1).

### Selection process

The selection of eligible studies followed the Population, Intervention, Comparison, Outcomes, Study Design (PICOS) principle. The eligibility of the searched studies was screened using a two-stage approach. First, the titles and abstracts were reviewed, followed by the full texts.

The initial screening process based on titles and abstracts was conducted with a stepwise approach as previously described [21]. When a searched study was found ineligible according to any of the exclusion criteria, it was immediately excluded without the consideration of other exclusion criteria. When the eligibility of a study was impossible to verify in the initial screening (e.g., some key information was not provided in the titles and abstracts), it would be included for full-text assessment. The study eligibility screening approach for the full-text assessment was the same as that used in the initial screening process. Reference lists of the searched systematic reviews and the studies selected for full-text assessment were also screened.

In order to identify studies that met the inclusion criteria, titles and abstracts of searched studies were first screened by two review authors (Leung WKC and Wong VCC), and then independently checked for relevancy by the other two coauthors (Cheung J and Lam SC), who are registered nurses having more than 10 years working in the nursing practice and education settings. The full text of these studies were retrieved and independently assessed for eligibility by the reviewers. Disagreements were resolved by discussion among the four reviewers.

### Data collection

Data about sedentary behavior were extracted from the included studies. Specifically, data, such as measurement methods (e.g., objectively-measured, self-reported, and observation), daily sedentary patterns (e.g., definitions, types, duration, and weekday-to-weekend variations), and participant characteristics (e.g., age, sample size, type of population [general or patient], body mass index [BMI] or body weight status [e.g., waist circumference and percent body fat], parity, and menopause conditions), were retrieved and presented. Sedentary time across studies was standardized in minutes per day for comparison. An assessment for the risk of bias or methodological quality of included evidence was generally not conducted within the scoping review because its aim was to shed light on an overview or map of the evidence [38]. Thus, the methodological quality appraisal of the included studies was not carried out in the present scoping review.

### Data synthesis

Similar to our previous scoping review [21], data for each study, including year of publication, country or region, age, sample size, and sedentary behavior patterns (types, definitions, measurements, and daily patterns), along with UI types, were retrieved and tabulated. A narrative synthesis of how the findings from the included trials were related to the study aims was carried out. All authors independently verified and edited every entry for data accuracy and consistency.

## Results

### Study selection

**Fig. 1:**
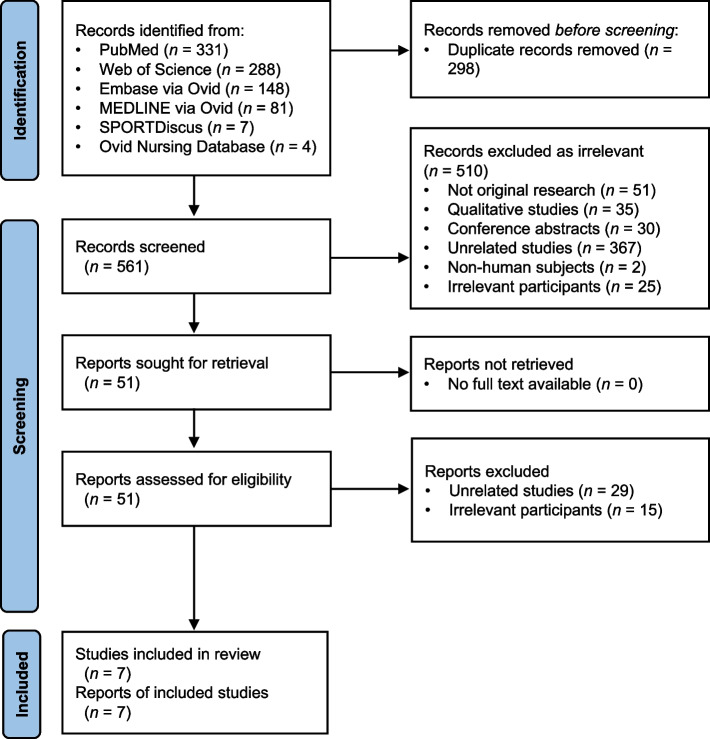
Study selection flow

Our electronic database search yielded a total of 859 records, of which 298 were duplicates. After the removal of duplicates, 561 studies were initially screened for titles and abstracts. The initial screening successfully included 51 potentially eligible studies for full-text assessment. After the full-text assessment, seven studies were finally included for review and reporting. Figure 1 demonstrated the study selection process.

### Study characteristics

Seven studies, namely, three experimental trials [39–41], three cross-sectional studies [4, 26, 42], and one secondary data analysis [25], were published between 2015 and 2023 and were conducted in four regions or countries, including Spain [4, 42], the United States [25, 40, 41], Malaysia [26], and Taiwan [39]. The sample sizes ranged from 33 to 712, with 1,822 female participants, and age varied from 64 to 97 years. The mean BMIs of the participants across the studies ranged from 25.4 kg·m^−2^ to 31.7 kg·m^−2^, which were categorized into overweight (25.0 kg·m^−2^ for non-Asians; 23.0 kg·m^−2^ for Asians) and obese (30.0 kg·m^−2^ for non-Asians; 25.0 kg·m^−2^ for Asians). Two nationally representative cross-sectional surveys showed that more than two-thirds of the participants (66.2%–85.9%) were overweight or obese [26, 42], of whom 63.5% had central obesity (waist circumference ≥ 88 cm) with percent body fat of 39.6% [42]. Two included studies was conducted merely in postmenopausal women [39, 42], of whom the average age of menopause was 49.0 years according to a cross-sectional study based on a randomly-selected, representative sample of noninstitutionalized older women [42].

### Patterns of sedentary behavior in older women with UI

Approximately 70.3% of older women having UI problems were sedentary (i.e., sitting most of the day), whereas 65.8% never engaged in any exercise [4]. Given that one of the included studies might have wrongly reported the sedentary time as they might have considered sleeping time (i.e., sedentary time exceeded 1000 min/day) [40], the sedentary behavior data measured by accelerometers derived from this study were not reported in the present review. Their objectively measured sedentary time varied from 493.3 to 508.4 min/day that accounted for around 73% of their waking time (Table 1) [25, 41], while self-reported sitting time on weekdays was 300–380 min/day (Table 2) [39, 40].

**Table 1:** Objectively measured sedentary behavior (*n* = 4)

Author, Country/Region, Study Design	Age (Years)		Participants			Sedentary Behavior			
	**Range**	**Mean / Median**	**Characteristics; Sample Size**	**Body Weight Status**	**Parity / Menopause Conditions**	**Type**	**Measurement**	**Definition**	**Daily Pattern**
Chu et al. (2021) [40]; United States; single-group pretest–posttest trial^a^	IQR: 71–81	76.5 ± 7.2	Women with overactive bladder; *n* = 75	BMI: - All ages: 31.7 ± 7.3 kg·m^−2^ - 65–69 years: 34.1 ± 9.0 kg·m^−2^ - 70–79 years: 33.9 ± 6.8 kg·m^−2^ - 80–89 years: 27.8 ± 4.9 kg·m^−2^ - ≥ 90 years: 23.4 ± 2.3 kg·m^−2^	n/a	Sedentary time and daily steps	ActiGraph GT3X	- ≤ 99 cpm - Daily steps: < 5000	All ages^b^: - Sedentary time: 1093.2 (1044.3–1167) min/day - %Time spent sedentary daily: 87.2 (82.8–92.5)% - Daily steps: 2117.75 (988.5–2924.3) 65–69 years: - Sedentary time: 1072.1 (1024.1–1158.3) min/day - %Time spent sedentary daily: 85.3 (80.9–91.9)% - Daily steps: 2716.1 (1190.4–3387.1) 70–79 years: - Sedentary time: 1092.9 (1032.6–1155.8) min/day - %Time spent sedentary daily: 87.2 (81.9–91.9)% - Daily steps: 2241.8 (1215.4–3089.3) 80–89 years: - Sedentary time: 1105.5 (1061.9–1169.9) min/day - %Time spent sedentary daily: 87.6 (84.7–92.6)% - Daily steps: 1088.1 (683.9–2798.8) ≥ 90 years: - Sedentary time: 1176.3 (1090.7–1195.5) min/day - %Time spent sedentary daily: 93.6 (86.6–94.6) % - Daily steps: 755.3 (526.9–1704.8)
Jerez-Roig et al. (2020) [26]; Malaysia; cross-sectional study	n/a	71.0 ± 8.0	- Total (UI and non-UI): *n* = 459 - Moderate-severe UI: *n* = 108 (23.5%) - Stress UI: *n* = 232 (50.5%) - Urge UI: *n* = 190 (41.4%)	BMI^c^: - Normal: 33.1% - Overweight: 30.7% - Obese: 29.0% - Extreme obese: 6.5%	n/a	Sedentary time	ActiGraph 7164; hip-worn; measurement: 7 consecutive days, waking time, considered valid if at least 5 days with 10 h of continuous wear time available	< 100 cpm	n/a
Chu et al. (2019a) [25]; United States; secondary data analysis	64–97	71	Women with UI; *n* = 35	BMI: - Median (Range): 30.4 (17.4–47.4) kg·m^−2^ - Mean (SD): 30.4 ± 7.5 kg·m^−2^	n/a	Sedentary time and daily steps	ActiGraph GT3X; waist-worn; measurement: considered valid given adequate accelerometer wear time as 4 days out of 7, with a minimum of 600 min (10 h) per day	- ≤ 99 cpm - Daily steps: < 5000	- Sedentary time: 493.3 (312.7–719.1) min/day - %Time spent sedentary daily: 73.9 (53.9–88.7)% - Daily steps: 2168.5 (686.8–5205.1) - Frequency of sedentary bouts: 11.3 (4.3–20.9) - Duration of sedentary bouts: 221.1 (22.4–436.9)
Chu et al. (2019b) [41]; United States; randomized trial	n/a	72.4 ± 6.3	Women with UI; *n* = 37	BMI: - Mean (Range): 26 (17.4—46.1) kg·m^−2^	n/a	Sedentary time and daily steps	ActiGraph GT3X; measurement: considered valid given adequate accelerometer wear time as 4 days out of 7, with a minimum of 600 min (10 h) per day	- ≤ 99 cpm - Daily steps: < 5000	- Sedentary time: 508.4 (410.5–553.2) min/day - %Time spent sedentary daily: 72.5 (67.7–76.0)% - Daily steps: 2671.7 (2168.5–3386.3) - Frequency of sedentary bouts: 11.3 (8.5–14.3) - Duration of sedentary bouts: 219.2 (169.7–311.0)

*Abbreviations*: *BMI* body mass index, *cpm* counts per minute, *IQR* interquartile range, *UI* urinary incontinence

^a^For experimental studies, only baseline data for either overall participants or participants in the experimental group were presented

^b^Data were presented in Median (Interquartile Range)

^c^BMI classification was according to the World Health Organization guidelines; normal (18.5–24.9 kg·m^−2^), overweight (> 25 kg·m^−2^), obesity (> 30 kg·m^−2^), and extreme obesity (≥ 40.0 kg·m^−2^)

**Table 2:** Self-reported sedentary behavior (*n* = 4)

Author, Country/Region, Study Design	Age (Years)		Participants			Sedentary Behavior			
	**Range**	**Mean / Median**	**Characteristics; Sample Size**	**Body Weight Status**	**Parity / Menopause Conditions**	**Type**	**Measurement**	**Definition**	**Daily Pattern**
Li et al. (2023) [39]; Taiwan; randomized trial^a^	n/a	66.1 ± 8.1	Postmenopausal women with UI; *n* = 33	BMI: 25.4 ± 5.6 kg·m^−2^	Parity: - Total number: 1.92 ± 1.26 - Vaginal delivery: 1.69 ± 1.32 - Caesarean section: 0.23 ± 0.83	Sitting time	IPAQ-short form	Time spent sitting on weekdays during the last 7 days	380 ± 158.4 min/weekday
Chu et al. (2021) [40]; United States; single-group pretest–posttest trial^a^	IQR: 71–81	76.5 ± 7.2	Women with overactive bladder; *n* = 75	BMI: - All ages: 31.7 ± 7.3 kg·m^−2^ - 65–69 years: 34.1 ± 9.0 kg·m^−2^ - 70–79 years: 33.9 ± 6.8 kg·m^−2^ - 80–89 years: 27.8 ± 4.9 kg·m^−2^ - ≥ 90 years: 23.4 ± 2.3 kg·m^−2^	n/a	Sitting time	IPAQ-long form	Time spent sitting on weekdays during the last 7 days	- All ages: 300 (60–720) min/weekday^b^ - 65–69 years: 330 (60–540) min/weekday - 70–79 years: 300 (60–720) min/weekday - 80–89 years: 360 (180–720) min/weekday - ≥ 90 years: 360 (180–360) min/weekday
Leirós-Rodríguez et al. (2017) [4]; Spain; cross-sectional study	n/a	78.8	Women with UI; *n* = 712	- BMI: 27 kg·m^−2^ - Weight: 70.1 kg	n/a	Sitting frequency	Which of these possibilities best describes your main activity during the day? (1) Sitting most of the day (2) Standing up most of the day without major displacements or efforts (3) I walk, I make frequent displacements (4) I perform tasks that require a great physical effort	Response (1): Sitting most of the day	70.3% of participants with UI were sedentary
Moreno-Vecino et al. (2015) [42]; Spain; cross-sectional study	66–91	74.6 ± 5.2	Non-institutionalized incontinent women; *n* = 134	- BMI^c^: 29.3 ± 4.3 kg·m^−2^ (a) Overweight: 46.0% (b) Obesity: 39.9% - Weight: 67.4 ± 10.2 kg - WC: 92.6 ± 11.7 cm (a) Central obesity^d^: 63.5% - Fat mass^e^: 39.6 ± 4.8% (a) Overweight: 41.4% (b) Obesity: 26.0%	Age of menopause: 49.0 ± 5.0 years	Sitting time	How many hours do you usually spend sitting per day?	Any activity in which the person had to be sitting (e.g., watching television, reading, sewing, etc.) at the present time	252 ± 78 min/day (including UI and non-UI)

*Abbreviations*: *BMI* body mass index, *IPAQ* International Physical Activity Questionnaire, *IQR* interquartile range, *UI* urinary incontinence, *WC* waist circumference

^a^For experimental studies, only baseline data for either overall participants or participants in the experimental group were presented

^b^Data were presented in Median (Interquartile Range)

^c^BMI classification was according to the World Health Organization guidelines; overweight (> 25 kg·m^−2^) and obesity (> 30 kg·m^−2^)

^d^Central obesity was defined according to the WHO criteria; ≥ 88 cm in women

^e^Overweight and obesity were defined according to the percent body fat of ≥ 38% and ≥ 43%, respectively

The step counts of the participants ranged from 2117.75 steps/day to 2671.7 steps/day (Table 1) [25, 40, 41], which were fewer than 5000 steps/day and therefore considered sedentary [21, 43, 44]. Notably, daily steps declined considerably with age from 2716.1 steps/day in individuals aged 65–69 years to 755.3 steps/day in those aged 90 years or older, but no obvious association was found between self-reported sitting time and advanced age [40].

### Association of sedentary behavior with UI outcomes

Using accelerometers, Jerez-Roig et al. [26] showed that a high percentage of time spent in sedentary behavior daily was observed in older women of any type versus aged-matched healthy counterparts. Moreover, the daily percentage of sedentary time was positively associated with moderately severe UI and urgency UI but not with stress UI. Adjusted for age and the number of comorbidities and vaginal deliveries, multivariate analyses demonstrated high mean duration of sedentary behavior bouts was associated with urgency UI but had no association with moderately severe UI or stress UI. However, neither objectively measured daily sedentary time nor self-reported sedentary behavior was correlated with any type of UI in the multivariate analyses.

Chu et al. [25] demonstrated that older women with UI who were the most sedentary had bothersome incontinence, increased use of incontinence products, and greater number of episodes of nocturia than those who were the least sedentary. Increase in the proportion of awake time spent sedentary was associated with increased number of nocturia episodes, whereas increased frequency of sedentary bouts was associated with increased use of incontinence products; however, these associations became insignificant after adjusted was made for daily step count.

Moreno-Vecino et al. [42] showed that there was no difference in self-reported sitting time between continent and incontinent women, and their mean sitting time was 258 min/day.

## Discussion

Gaining an improved understanding of sedentary behavior patterns in older people and clinical populations is essential because a high frequency of daily sedentary behavior is associated with heightened risk of mortality and developing many chronic and debilitating conditions [18]. In this scoping review, the objective measurement of sedentary behavior showed that noninstitutionalized older women with UI spent more than 8 h (493.3–508.4 min/day) daily on sedentary behavior equivalent to 73% of their awake time, which was greater than age-matched healthy counterparts. This objectively measured sedentary time was comparable to that reported in a systematic review that examined the patterns of sedentary behavior in community-dwelling older people aged 60 + years (65%–80% of waking hours) [28]. Similar to other reviews [21, 28], older women with UI self-reported a lower amount of daily sitting times (300–380 min/weekday).

This scoping review is the first to suggest a potential link between increased amounts of sedentary behavior and prevalence of UI in noninstitutionalized older women. We reported that greater sedentary time is associated with increased severity and a particular type of UI (i.e., urgency UI but not stress UI) [26]. After adjustment for advanced age, comorbidities, and number of vaginal deliveries, which are known risk factors to UI [45], the average duration of sedentary behavior bouts was positively associated with urgency UI. This finding was consistent with recent research, which showed that interrupting prolonged bouts of sedentary time is favorably correlated with health outcomes (e.g., central obesity) in older women, but not in older men, independent of total sedentary time [46]. The lowered likelihood of suffering from abdominal obesity, hence reducing excess weight and pressure in the abdominal area and bladder, can explain the relationship between breaking up prolonged sedentary behavior bouts and UI occurrence.

Sedentary behavior is positively associated with UI outcomes, such as bothersome incontinence and use of incontinence products, in older women with UI, but the associations between sedentary time or bouts and such UI outcomes were attenuated after adjustment for daily step counts [25]. Ekelund et al. [47] revealed that high levels of physical activity equate to 60–75 min/day of moderate-intensity physical activity daily and are effective in attenuating increased risk of mortality associated with prolonged sitting and high TV-viewing time. The 2011 compendium of physical activities indicated that walking at a slow-to-moderate pace (2–3.2 mph) on a firm surface varied from 2.8 METs to 3.5 METs [48]. Given age-related decline in maximal aerobic capacity, older and young adults working at the same absolute MET level usually have varied relative levels of physical exertion. In other words, despite having the same MET level as young adults, older adults are working at a relatively higher maximal rate of oxygen consumption, achieving moderate-intensity aerobic physical activity levels even for slow walking. Brisk walking (3 mph) confers cardiorespiratory endurance, enhances muscle strength, and results in favorable body composition in healthy older individuals, and high-intensity brisk walking is effective in improving their aerobic capacities [49]. In a meta-analysis of 15 international cohorts, a dose–response association between increased number of daily steps and reduced risks of all-cause mortality was apparently evident in adults aged 60 years or older, and the hazard ratio of mortality plateau was reached at 6000–8000 steps per day [50]. However, given that the included studies primarily examining the associations of sedentary behavior with UI outcomes were all cross-sectional in nature [25, 26, 42], the relationship between a sedentary lifestyle and UI outcomes was apparently bidirectional (i.e., no indication for any temporal, cause-and-effect relationships). In other words, whether people who are more sedentary have greater UI risks or whether people who have UI are more sedentary remains largely unknown. More rigorous large-scale studies, especially randomized controlled trials, are imperatively needed to evaluate the intricate relationships among sedentary behavior, physical activity (especially daily steps), and UI.

Questionnaire-based instruments may not be an accurate measure for examining sedentary behavior in older adults largely because of their cognitive-reliant properties and social desirability bias. For the Sedentary Behavior Questionnaire, older people are required to select the amount of time specifically spent on nine common sedentary behaviors on a typical weekday or a weekend day. For the International Physical Activity Questionnaire, participants will be asked to specify the amount of time they spend sitting on a weekday or a weekend day during the last seven days. The criterion validity (i.e., how accurately a test measures the outcome it is primarily intended to examine) of self-reported measures for older people who generally have age-associated memory decline is largely questionable, partly explaining the absence of significant differences in self-reported sitting time between continent and incontinent women [42]. Social desirability bias can explain why self-reported sitting time is usually lower than objectively-measured sedentary time [39, 40, 42]. Therefore, an accelerometer (e.g., ActivPAL for distinguishing upright and non-upright positions) seems to be a more valid measuring tool for sedentary behavior than questionnaires in older populations. Chu et al. [25] reported that the majority (85.7%) of older women with UI (31% of them had neurocognitive dysfunction) were able to provide valid accelerometer data defined as that collected for four or more days of wear time per week with 10 h/day or more. The provision of accurate accelerometer-based data can aid in the development of exercise clinical trials and thus increase treatment options for UI.

However, insights gained from this scoping review are limited for several reasons. First, given that few studies examined the patterns of sedentary behavior in women with UI, we were unable to report the weekday-to-weekend trends in sedentary behavior and the context in which the sedentary time was commonly accrued (e.g., TV watching, computer use, socializing, sitting, and talking on the phone). Second, the temporal, causal relationship between sedentary behavior and UI outcomes in older women cannot be assertively confirmed because of the limited amount of relevant evidence, which was obtained from nonexperimental observational studies in which data about sedentary behavior and UI variables were collected at a single point in time [25, 26, 42]. Third, owing to a small number of relevant studies, cross-country or -region comparisons cannot be made, which are of utmost importance because they facilitate the identification of needed improvements and the formulation of recommendations derived from countries or regions with excellent perform in the sedentary behavior domain. Fourth, owing to the heterogeneity of methods for measuring sedentary behavior, a comprehensive meta-analytic comparison between continent and incontinent women was not conducted. Lastly, we did not involve qualified librarians in the electronic database search. Given that the first author (Leung WKC) had previous experience of conceiving and conducting scoping or systematic review research [21, 36, 51], we believe that our search strategy for electronic databases was trustworthy and sensitive enough for comprehensive coverage of the available literature as exemplified by a retrieval of more than 800 records by our electronic database search and only 0.8% (*n* = 7) included for review and reporting. Moreover, previous studies showed that electronic database search using PubMed and EMBASE covered up to 91% of the published literature [52, 53]. Apart from PubMed and EMBASE, four more electronic databases, namely Web of Science, SPORTDiscus, Ovid Nursing Database, and MEDLINE, were employed in the present scoping review to ensure breadth of coverage.

## Conclusion

Depending on reporting methods, older women with UI on average spend 5–8 h/day on sedentary activities, and this duration is generally greater than that of age-matched non-UI counterparts. With the improved knowledge about the relationships between sedentary behavior and UI occurrence, further clinical studies using large sample sizes and intervening daily sedentary time are of great importance to public health.

### Supplementary Information

**Supplementary Material 1.**

## Data Availability

Not applicable.

## Abbreviations

BMI: Body mass index
MET: Metabolic equivalent of task
PICOS: Population, Intervention, Comparison, Outcomes, Study Design
PRISMA: Preferred Reporting Items for Systematic Reviews and Meta-Analyses
TV: Television
UI: Urinary incontinence
