# Supplementary material for: Patterns of sedentary behavior among older women with urinary incontinence and urinary symptoms: a scoping review
Source: BMC Public Health. 2024 Apr 30;24:1201. doi: 10.1186/s12889-024-18703-7 (PMC11059602; doi:10.1186/s12889-024-18703-7)
Supplement: Supplementary file 1 — Supplementary Material 1. [file 12889_2024_18703_MOESM1_ESM.docx]

**Additional file 1**

**Electronic Database Search Strategies**

1. **PubMed Search Strategy**

(("urinary incontinence"[All Fields] OR "bladder incontinence"[All Fields] OR ("enuresis"[MeSH Terms] OR "enuresis"[All Fields]) OR ("nocturnal enuresis"[MeSH Terms] OR ("nocturnal"[All Fields] AND "enuresis"[All Fields]) OR "nocturnal enuresis"[All Fields] OR ("bed"[All Fields] AND "wetting"[All Fields]) OR "bed wetting"[All Fields])) AND ("sedentar*"[All Fields] OR "tv viewing"[All Fields] OR "tv watching"[All Fields] OR ("televised"[All Fields] OR "televising"[All Fields] OR "television"[MeSH Terms] OR "television"[All Fields] OR "televisions"[All Fields] OR "television s"[All Fields]) OR ("smartphone"[MeSH Terms] OR "smartphone"[All Fields] OR "smartphones"[All Fields] OR "smartphone s"[All Fields]) OR ("sitting position"[MeSH Terms] OR ("sitting"[All Fields] AND "position"[All Fields]) OR "sitting position"[All Fields] OR "sitting"[All Fields] OR "sittings"[All Fields]) OR ("reclination"[All Fields] OR "recline"[All Fields] OR "reclined"[All Fields] OR "recliner"[All Fields] OR "reclining"[All Fields]) OR ("supine position"[MeSH Terms] OR ("supine"[All Fields] AND "position"[All Fields]) OR "supine position"[All Fields] OR "lying"[All Fields] OR "deception"[MeSH Terms] OR "deception"[All Fields]))) AND ((fft[Filter]) AND (1000/1/1:2023/3/31[pdat]) AND (english[Filter]))

**Limiters**

- Text availability: Full text
- Publication date: From 1000/1/1 to 2023/5/31
- Language: English

1. **Web of Science Search Strategy**

All Fields = "urinary incontinence" OR "bladder incontinence" OR enuresis OR bed?wetting

AND

All Fields = sedentar* OR "tv viewing" OR "tv watching" OR television OR smartphone OR sitting OR reclining OR lying

**Limiters**

- Document Types: Articles
- Language: English

1. **SPORTDiscus Search Strategy**

("urinary incontinence" OR "bladder incontinence" OR enuresis OR bed?wetting)

AND

(sedentar* OR "tv viewing" OR "tv watching" OR television OR smartphone OR sitting OR reclining OR lying)

**Limiters**

- Full text
- Language: English

1. **Ovid Nursing Database Search Strategy via Ovid**

| 1. | urinary incontinence.mp. or exp Urinary Incontinence/ |
| --- | --- |
| 2. | bladder incontinence.mp. |
| 3. | enuresis.mp. or exp Enuresis/ |
| 4. | bedwetting.mp. |
| 5. | bed-wetting.mp. |
| 6. | sedentary.mp. |
| 7. | sedentariness.mp. |
| 8. | tv viewing.mp. |
| 9. | tv watching.mp. |
| 10. | television.mp. or exp Television/ |
| 11. | smartphone.mp. |
| 12. | sitting.mp. or exp Sitting/ |
| 13. | reclining.mp. |
| 14. | lying.mp. |
| 15. | 1 or 2 or 3 or 4 or 5 |
| 16. | 6 or 7 or 8 or 9 or 10 or 11 or 12 or 13 or 14 |
| 17. | 15 and 16 |
| 18. | limit 17 to (english language and full text) |

**Limiters**

- Full Text
- English Language

1. **EMBASE Search Strategy via Ovid**

| 1. | urinary incontinence.mp. or urine incontinence/ |
| --- | --- |
| 2. | bladder incontinence.mp. |
| 3. | enuresis.mp. or enuresis/ |
| 4. | bedwetting.mp. |
| 5. | bed-wetting.mp. |
| 6. | sedentar*.mp. |
| 7. | tv viewing.mp. or television viewing/ |
| 8. | tv watching.mp. |
| 9. | television.mp. or television/ |
| 10. | smartphone.mp. or smartphone/ |
| 11. | sitting.mp. or sitting/ |
| 12. | reclining.mp. |
| 13. | lying.mp. |
| 14. | 1 or 2 or 3 or 4 or 5 |
| 15. | 6 or 7 or 8 or 9 or 10 or 11 or 12 or 13 |
| 16. | 14 and 15 |
| 17. | limit 16 to (full text and english language) |

**Limiters**

- Full Text
- English Language

1. **MEDLINE Search Strategy via Ovid**

| 1. | urinary incontinence.mp. or Urinary Incontinence/ |
| --- | --- |
| 2. | bladder incontinence.mp. |
| 3. | Enuresis/ or enuresis.mp. |
| 4. | bedwetting.mp. |
| 5. | bed-wetting.mp. |
| 6. | sedentar*.mp. |
| 7. | tv viewing.mp. |
| 8. | tv watching.mp. |
| 9. | television.mp. or Television/ |
| 10. | smartphone.mp. or Smartphone/ |
| 11. | sitting.mp. |
| 12. | reclining.mp. |
| 13. | lying.mp. |
| 14. | 1 or 2 or 3 or 4 or 5 |
| 15. | 6 or 7 or 8 or 9 or 10 or 11 or 12 or 13 |
| 16. | 14 and 15 |
| 17. | limit 16 to (english language and full text) |

**Limiters**

- Full Text
- English Language
